# Citrullinated Epitopes Identified on Tumour MHC Class II by Peptide Elution Stimulate Both Regulatory and Th1 Responses and Require Careful Selection for Optimal Anti-Tumour Responses

**DOI:** 10.3389/fimmu.2021.764462

**Published:** 2021-11-09

**Authors:** Peter Symonds, Ana Marcu, Katherine W. Cook, Rachael L. Metheringham, Lindy G. Durrant, Victoria A. Brentville

**Affiliations:** ^1^ Scancell Limited, Biodiscovery Institute, University of Nottingham, Nottingham, United Kingdom; ^2^ Department of Immunology, Interfaculty Institute for Cell Biology, University of Tübingen, Tübingen, Germany; ^3^ Cluster of Excellence iFIT (EXC 2180) “Image-Guided and Functionally Instructed Tumour Therapies”, University of Tübingen, Tübingen, Germany; ^4^ Biodiscovery Institute, Division of Cancer and Stem Cells, University of Nottingham, Nottingham, United Kingdom

**Keywords:** CD4 T cell, citrulline, cancer immunotherapy, peptide elution, post-translational modification (PTM)

## Abstract

**Background:**

Somatic mutations or post-translational modifications of proteins result in changes that enable immune recognition. One such post-translational modification is citrullination, the conversion of arginine residues to citrulline. Citrullinated peptides are presented on MHC class II (MHCII) *via* autophagy which is upregulated by cellular stresses such as tumourigenesis.

**Methods:**

Peptides were eluted from B16 melanoma expressing HLA-DP4 and analysed by mass spectrometry to profile the presented citrullinated repertoire. Initially, seven of the identified citrullinated peptides were used in combination to vaccinate HLA-DP4 transgenic mice. Immune responses were characterised from the combination and individual vaccines by *ex vivo* cytokine ELISpot assay and assessed for tumour therapy.

**Results:**

The combination vaccine induced only weak anti-tumour therapy in the B16cDP4 melanoma model. Immune phenotyping revealed a dominant IFNγ response to citrullinated matrix metalloproteinase-21 peptide (citMMP21) and an IL-10 response to cytochrome p450 peptide (citCp450). Exclusion of the IL-10 inducing citCp450 peptide from the combined vaccine failed to recover a strong anti-tumour response. Single peptide immunisation confirmed the IFNγ response from citMMP21 and the IL-10 response from citCp450 but also showed that citrullinated Glutamate receptor ionotropic (citGRI) peptide stimulated a low avidity IFNγ response. Interestingly, both citMMP21 and citGRI peptides individually, stimulated strong anti-tumour responses that were significantly better than the combined vaccine. In line with the citGRI T cell avidity, it required high dose immunisation to induce an anti-tumour response. This suggests that as the peptides within the combined vaccine had similar binding affinities to MHC-II the combination vaccine may have resulted in lower presentation of each epitope and weak anti-tumour immunity.

**Conclusion:**

We demonstrate that tumours present citrullinated peptides that can stimulate Th1 and regulatory responses and that competition likely exists between similar affinity peptides. Characterisation of responses from epitopes identified by peptide elution are necessary to optimise selection for tumour therapy.

## Introduction

Progress made in immune-oncology has demonstrated a role for the immune system in the surveillance of cancer (1). Recently this has focussed in part on the identification and targeting of somatic mutations that form neoantigens (2, 3). These neoantigens can be targeted by various approaches for tumour therapy (4–6). The identification of mutated neoantigen targets from next generation sequencing data involves the use of prediction algorithms for MHC binding but may not take into account whether the epitopes are naturally presented. Prediction algorithms are more robust for MHC class I (MHCI) binding peptides thus, the majority of effort has been focussed upon the identification of T cell responses to CD8 T cell epitopes presented *via* MHCI alleles. The identification of MHC class II (MHCII) binding epitopes is more difficult since MHC peptide binding prediction algorithms are less reliable. In addition to this, the processing and presentation of the MHC class II epitopes from antigens can occur *via* different pathways, two of which are phagocytosis and autophagy (7) which further increases epitope diversity. A number of recent studies have demonstrated an important role for CD4 T cells in tumour control (8–11). Therefore, more work is being undertaken to identify target tumour epitopes for CD4 T cells.

Mass spectrometry based immunopeptidomics has the potential to identify tumour-derived epitopes that are naturally presented by MHC molecules (12, 13) and has been used in combination with prediction algorithms to facilitate the selection of potential epitopes (14–16). In addition to identification of tumour-associated epitopes and mutated neo-epitopes, mass spectrometry also has the capability to identify post-translationally modified epitopes. These epitopes cannot be identified through next generation genome sequencing or predicted using algorithms as they are not encoded in the genomic DNA or present in RNA transcripts and current MHC binding algorithms do not take into account any post-translational modifications. One such post-translational modification is citrullination, the conversion of arginine to citrulline by a family of peptidylarginine deaminase (PAD) enzymes.

Citrullination can occur as a result of the degradation and recycling process known as autophagy that is induced in stressed cells (17), including cancer cells. Indeed in breast cancer levels of PAD expression have been shown to correlate with increased MHCII bound citrullinated peptides (18). The detection of CD4 T cells specific for citrullinated peptides also suggests that these post-translationally modified epitopes are presented on MHCII molecules. Indeed we and others have shown that citrullinated peptides presented *via* autophagy can be targets for CD4 T cells (17, 19). In the presence of inflammation, citrullinated epitopes can be presented by MHCII molecules on tumours for recognition by CD4 T cells. We have shown that the potent immune responses unleashed in response to citrullinated epitopes can be harnessed and redirected to destroy cancer cells (11, 19, 20). The identification of citrullinated peptides that may be presented on tumour cells is challenging since MHCII binding predictions do not accommodate provision for this post-translational modification and the citrulline modification can influence binding of the peptide to MHCII (20, 21). In this study we have used peptide elution and mass spectrometry for the identification of potential citrullinated epitopes using the mouse B16 melanoma model in HLA-DP4 transgenic mice. We identify a number of citrullinated peptides of which only a subset stimulate immune responses in mice. Three presented peptides elicit phenotypically distinct immune responses with those eliciting a Th1 response delivering efficient anti-tumour therapy, which is dramatically reduced when these are included in a combination vaccine of multiple eluted peptides. We provide evidence of an IL-10 producing response to a citrullinated peptide presented by B16 tumours suggesting regulatory T cell repertoires exist to citrullinated peptides. Our results suggest that identification of citrullinated epitopes for cancer therapy by peptide elution and mass spectrometry can yield appropriate immunotherapeutic targets, however care should be taken when designing combinations of eluted peptides for immunotherapy regimes.

## Materials and Methods

### Peptide Identification by Elution From Tumours and Mass Spec Analysis

Tumour cells were implanted into HLA-DP4 transgenic mice and tumours excised once they reached >10mm in diameter. Tumours were dissociated and homogenized in lysis buffer consisting of 10 mM CHAPS (Panreac AppliChem) and one cOmpleteTM protease inhibitor cocktail tablet (Roche) in PBS. HLA-DP4 molecules were isolated from disaggregated tumour samples through immunoaffinity purification employing the pan-HLA-II-specific antibody Tü39 produced in house (22) covalently linked to CNBr-activated sepharose (Sigma-Aldrich). The tumour lysate was passed over the affinity columns cyclically overnight at 4°C. Peptides were eluted with 0.2% TFA and subsequently separated from HLA molecular remnants by ultracentrifugation employing 10 kDa Amicon filter units (Merck Millipore). The eluate volume was reduced to approximately 50 µl by lyophilization, and the reduced peptide solution was purified and desalted in five times using ZipTip pipette tips with C18 resin and 0.6 µl bed volume (Merck Millipore) and eluted in 32.5% ACN/0.2% TFA. Peptide fragments were then concentrated by vacuum centrifugation and supplemented with 1% ACN/0.05% TFA for mass spectrometric analysis.

Peptides were separated by reversed-phase liquid chromatography (nanoUHPLC, UltiMate 3000 RSLCnano; Dionex) and analyzed in an online-coupled LTQ Orbitrap XL (Thermo Fisher Scientific) mass spectrometer. Three technical replicates, 5 µl each, per sample were injected onto a 75 µm x 2cm trapping column (Acclaim PepMap RSLC; Dionex) at 4 µl/min for 10 min. Peptide separation was performed at 50°C and a flow rate of 175 nl/min on a 50 µm x 25 cm separation column (Acclaim PepMap RSLC; Dionex), applying a gradient ranging from 3 to 30% solvent B over the course of 90 min (solvent A: 99% H2O, 1% acetonitrile and 0.1% formic acid; solvent B: 20% H2O, 80% acetonitrile and 0.04% formic acid). Eluting peptides were analyzed on an LTQ Orbitrap XL mass spectrometer using a top five CID (collision-induced dissociation) fragmentation method. The mass range was limited to 300–1500 m/z with charge states 2+ to 5+ selected for fragmentation. Precursor ions were detected in the Orbitrap at a resolution of 60,000, while fragment ions were detected in the linear ion trap. The dynamic exclusion was set to 3 seconds and the mass tolerance set to +/- 10 ppm.

MS data analysis was performed using the Sequest search algorithm (23) embedded in the Proteome discoverer 1.4 (Thermo Fisher). Fragment spectra were searched against the murine proteome as comprised in the UniProtKB/Swiss-Prot database (www.uniprot.org released 29.05.2015, 16,716 protein sequences) followed by FDR estimation using the percolator algorithm (5% threshold). No cleavage specificity was selected, and methionine oxidation and arginine deamination (citrullination) were set as dynamic modifications. The maximal delta Cn was set to 0.05, the precursor mass tolerance to +/- 5ppm, the fragment mass tolerance to +/- 0.5 Da. The mass spectrometry proteomics data have been deposited to the ProteomeXchange Consortium *via* the PRIDE (24) partner repository with the dataset identifier PXD029029.

### Peptides

Synthetic citrullinated peptides Glutamate receptor ionotropic 316-340 (citGRI) EPKSSCYNTHEK-cit-IYQSNMLN-cit-YLI, Trophoblast glycoprotein-like protein 40-61 (cit5T4) ML-cit-CASGAEL-cit-QPPRDVPPDAR, Cytochrome P450 1A2 373-392 (citCp450) LEIY-cit-YTSFVPFTIPHSTTR, Matrix metalloproteinase-21 337-360 (citMMP21) VFDWIRKERNQYGEVRV-cit-FNTYFFR, Serine/threonine-protein phosphatase 2A 55 kDa regulatory subunit B gamma isoform 370-391 (citSeThrePho) KRDVTLEAS-cit-ESSKP-cit-AVLKP-cit-, 26S protease regulatory subunit 10B 246-268 (cit26S-10B) MDEIDAIGG-cit-RFSEGTSADREIQ and Elongation factor 1-alpha 1 130-154 cit (citELO) NGQT-cit-EHALLAYTLGVKQLIVGVNK and synthetic native peptides Glutamate receptor ionotropic 316-340 EPKSSCYNTHEKRIYQSNMLNRYLI (GRI), Cytochrome P450 1A2 373-392 LEIYRYTSFVPFTIPHSTTR (Cp450), Matrix metalloproteinase-21 337-360 VFDWIRKERNQYGEVRVRFNTYFFR (MMP21) were used in these assays. Peptides were manufactured by Genescript, USA and stored lyophilised at -80°C until use.

### Cell Lines

The B16F1 cell line has been knocked out for murine MHC class I and II (B16MHKO) by Zinc finger Technology (Sigma Aldrich, UK) and engineered to express HLA-DP4 under a constitutive (cDP4) or IFNγ inducible (iDP4) promoter and HHDII as described previously (20).

### HLA- DP4 Binding Studies

Binding of peptides to HLA-DP4 was assessed by extraction of membrane fractions from B16HHDII/cDP4 cells using Mem-PER™ Plus Membrane Protein Extraction Kit (Thermo Fisher-Scientific) according to manufacturer’s instructions. Membrane preps containing HLA-DP4 were incubated with 10 µg biotinylated peptide for 4hrs at 37°C. For competition assays, the membrane preps and 10 µg biotinylated Hepatitis B peptide were pre-incubated in the presence of specified concentrations of non-biotinylated peptides. Biotinylated peptide bound to HLA-DP4 was detected by capture onto streptavidin-coated ELISA plates and detected with anti-HLA-DP antibody clone B7/21 (Leinco Technologies Inc, USA) and anti-mouse IgG3 HRP antibody (Invitrogen, UK). Binding was quantified with TMB substrate and absorbance read at 405nm wavelength.

### Mice and Immunisations

Animal work was carried out under a Home Office approved project license at Nottingham Trent University using mice aged between 8-20 weeks. HLA-A2.1+/+ HLADP4+/+ hCD4+/+ (HHDII/DP4) transgenic mice (EM:02221, European Mouse Mutant Archive) express human CD4 in replacement of murine CD4 and are deficient in both murine MHC class I and II. Murine MHC are replaced with the chimeric HLA-A2 (HHDII) and human DP4 alleles. HHDII/DR1 mice (Pasteur Institute, France) are deficient in both murine MHC class I and II and murine MHC are replaced with the chimeric HLA-A2 (HHDII) and human DR1 alleles.

For all studies, the mice were randomised into different groups but not blinded to the investigators. Peptides were dissolved in PBS and then emulsified with CpG (ODN 1826, Invivogen) and MPLA (Invivogen) and delivered at 10 or 25µg dose unless stated otherwise. Adjuvants were used at 5µg/dose. Peptides in adjuvant were injected subcutaneously at the base of the tail. Mice were immunised on day 1, 8 and 15 and spleens were removed for analysis at day 21.

For the tumour therapy studies, HHDII/DP4 transgenic mice were challenged by subcutaneous injection with 5 × 105 B16/HHDII/cDP4 (B16cDP4) cells or 1 × 105 B16/HHDII/iDP4 (B16iDP4) cells subcutaneously on the flank at day 1 and then immunised on days 4, 11 and 18. Tumour growth was monitored at 3–4 days intervals and mice were humanely euthanised once tumour approached 15mm diameter.

### *Ex Vivo* Elispot Assay

ELISpot assays were performed using murine IFNγ and IL-10 capture and detection reagents according to the manufacturer’s instructions (Mabtech). In brief, the specific cytokine antibodies were coated onto wells of 96-well Immobilin-P plates. Synthetic peptides (Genescript) were used at 10 µg/mL and diluted in culture media (RPMI medium 1640 (GIBCO/BRL) supplemented with 10% FCS (Sigma), 2mM L-glutamine (Sigma) and sodium bicarbonate buffered with additional 20mM HEPES (Sigma) and 50 µM 2-mercaptoethanol (2ME, Thermofisher). 5x10^5^/well splenocytes were added in quadruplicate wells, plates were incubated for 40 hours at 37˚C/5% CO_2_. Cells with culture media only were added as negative control and cells cultured with 5 µg/ml Lipopolysaccharide (LPS, Sigma) were used as a positive control. Where relevant anti-CD4 (human clone OKT4, BioXcell) or CD8 (mouse clone 2.43, BioXcell) blocking antibodies were added to splenocytes at 20µg/ml for 15 minutes prior to stimulation with peptide. After incubation, captured cytokine was detected by biotinylated cytokine specific antibody and developed using streptavidin alkaline phosphatase and chromogenic substrate. Spots were analysed and counted using an automated plate reader (Cellular Technologies Ltd). Results were represented as spots/million splenocytes.

### Statistical Analysis

Comparative analysis of the Elispot assay data and peptide binding assays were performed by applying paired 2 tailed student’s t test or ordinary one way ANOVA with sidak’s multiple comparisons test and values of p calculated accordingly. Comparison of tumour survival was assessed by Log Rank test using Graphpad Prism 8 software (GraphPad Software, Inc.). p < 0.05 was considered statistically significant.

## Results

### Identification of Citrullinated Peptides Presented on Tumour Associated MHC Class II

Citrullinated peptides can be presented by tumour cells in association with MHCII molecules. The presentation of citrullinated peptides has been shown for several MHCII alleles and we have previously shown the presentation through HLA-DP4 allele (20). We therefore undertook a discovery approach to identify other citrullinated epitopes presented by tumour cells using the mouse B16F1 tumour model expressing human HLA-DP4. By isolating MHCII ligands from these tumours through immunoaffinity chromatography, we have identified 385 peptides from three replicate analyses employing a 5% FDR threshold (**Supplementary Tables 1**–**3**). Of these, 25 peptide sequences containing citrulline were identified with high or medium confidence (**Table 1**).

**Table 1:** Sequences identified by mass spectrometry.

Peptide sequence	Protein identification	Protein accession number	Gene name
ALLLRVYAxGxKQ	Adenosine 3’-phospho 5’-phosphosulfate transporter 1	Q91ZN5	Slc35b2
LPPGIRWPxRNRSSLRRRWLHH	WSC domain-containing protein 1	Q80XH4	Wscd1
KVKLVVxYTPKVLEEMESR	Protein lin-7 homolog C	O88952	Lin7c
AQREEExSQADSALYQMQLETEKER	Centrosome-associated protein CEP250	Q60952	Cep250
TVEDxFDQQKNDYDQLQKA	Desmoplakin	E9Q557	Dsp
LEIYxYTSFVPFTIPHSTTR	Cytochrome P450 1A2	P00186	Cyp1a2
VAIAxAIL	ATP-binding cassette sub-family B member 7, mitochondrial	Q61102	Abcb7
NGQTxEHALLAYTLGVKQLIVGVNK	Elongation factor 1-alpha 1	P10126	Eef1a1
GMExVWCVASLxGSNNVALGYDE	Coatomer subunit beta’	O55029	Copb2
VPxHPAATSWYEEFxRLYDMVPCV	F-box/WD repeat-containing protein 5	Q9QXW2	Fbxw5
MLxCASGAELxQPPRDVPPDAR	Trophoblast glycoprotein-like	Q8C013	Tpbgl
IKxCSEFESxLEGYSKELEMFRKRE	Dynein heavy chain 3, axonemal	Q8BW94	Dnah3
YLLPILVLFLAYYYYSTNEEFx	Corticosteroid 11-beta-dehydrogenase isozyme 1	P50172	Hsd11b1
FxTIHQACKLICxK	Zinc finger and BTB domain-containing protein 8A	Q9CWH1	Zbtb8a
GxHNGIDGLIPHQYIVVQDTEDG	SLIT-ROBO Rho GTPase-activating protein 2	Q91Z67	Srgap2
ExKRARGISPIVF	YTH domain-containing protein 1	E9Q5K9	Ythdc1
VFDWIRKERNQYGEVRVxFNTYFFR	Matrix metalloproteinase-21	Q8K3F2	Mmp21
LPECIDALTVSLESVQSxAAWR	Nesprin-2	Q6ZWQ0	Syne2
MQRMHNPEREAKKADxISRSKTF	PR domain zinc finger protein 10	Q3UTQ7	Prdm10
QKTGTAEMSSILEExILGADTSVD	ATP synthase subunit alpha, mitochondrial	Q03265	Atp5f1a
LGWSAPVAISRPLxACQM	Ubiquitin carboxyl-terminal hydrolase 37	Q8C0R0	Usp37
EPKSSCYNTHEKxIYQSNMLNxYLI	Glutamate receptor ionotropic, NMDA 2B	Q01097	Grin2b
FLSCFSPEYRxITL	Synaptic vesicle glycoprotein 2A	Q9JIS5	Sv2a
KRDVTLEASxESSKPxAVLKPx	Serine/threonine-protein phosphatase 2A 55 kDa regulatory subunit B gamma isoform	Q8BG02	Ppp2r2c
MDEIDAIGGxRFSEGTSADREIQ	26S protease regulatory subunit 10B	P62334	Psmc6

x, citrulline.

Despite the citrullinated versions being eluted from HLA-DP4 on tumour cells, analysis of all native sequences showed a range of predicted binding scores using the IEDB predictions software for binding to HLA-DP4 (**Supplementary Table 4**). Citrulline modification can influence both the binding affinity to MHCII and the contact with the TCR (25) although the citrulline modification is not taken into account in binding prediction software. In order to select a smaller number of peptides to test, we chose 7 peptides that all contained the detected citrulline modification(s) within the predicted native core binding region but which showed a range of predicted binding scores based upon the native sequence (**Table 2**). The MHCII binding predictions were made on 5/20/2020 using the IEDB analysis resource Consensus tool (26, 27).

**Table 2:** Sequences chosen for further analysis.

Peptide sequence	Length (aa)	Protein & coordinates	Predicted HLA-DP4 binding score	Predicted core binding region
NGQTxEHALLAYTLGVKQLIVGVNK	24	Elongation factor 1-alpha 1 (aa130-154)	26.00	T**R**EHALLAY
KRDVTLEASxESSKPxAVLKPx	21	Serine/threonine-protein phosphatase 2A 55 kDa regulatory subunit B gamma isoform (aa370-391)	65.50	VTLEAS**R**ES LEAS**R**ESSK
MDEIDAIGGxRFSEGTSADREIQ	22	26S protease regulatory subunit 10B (aa246-268)	56.00	IGG**R**RFSEG
EPKSSCYNTHEKxIYQSNMLNxYLI	24	Glutamate receptor ionotropic, NMDA 2B (aa316-340)	31.0	K**R**IYQSNML YQSNMLN**R**Y
MLxCASGAELxQPPRDVPPDAR	21	Trophoblast glycoprotein-like (aa40-61)	61.5	L**R**CASGAEL
LEIYxYTSFVPFTIPHSTTR	19	Cytochrome P450 1A2 (aa373-392)	0.41	IY**R**YTSFVP Y**R**YTSFVPF
VFDWIRKERNQYGEVRVxFNTYFFR	23	Matrix metalloproteinase-21 (aa337-360)	17.0	RV**R**FNTYFF EVRV**R**FNTY

x = citrulline, bold = arginine that is citrullinated.

The MHCII binding predictions were made on 5/20/2020 using the IEDB analysis resource Consensus tool (26, 27).

### Vaccination With a Combination Peptide Vaccine Provides Poor Tumour Therapy

**Figure 1:**
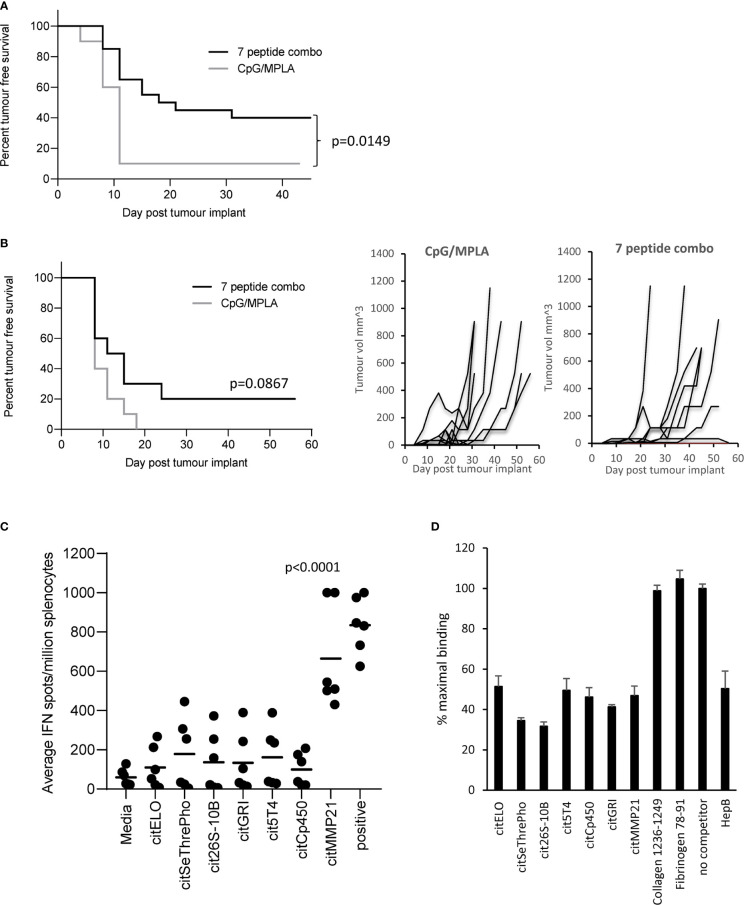
Citrullinated peptide combination vaccination provides poor tumour therapy in HLA-DP4 transgenic mice. HLA-DP4 transgenic mice were challenged with B16 cells constitutively expressing DP4 **(A)** or expressing DP4 under an IFNγ inducible promoter **(B)** and four days later mice were immunised with combination of 10ug each of seven eluted peptides and tumour growth and survival monitored. N=10/group. **(C)**, HLA-DP4 transgenic mice were immunised with seven peptide combination vaccine at days 1, 8 and 15 and immune responses monitored at day 21 by IFNγ ELISpot assay. Results are representative of at least two independent experiments in which n=3. **(D)**, Competition of equal quantity of non-biotinylated competitor peptides in the presence of 10µg biotinylated HepB 181-193 peptide. Results are representative of at least two independent experiments.

Vaccination with the 7 selected peptide combination was screened to see if it could provide tumour therapy in the B16 tumour model constitutively expressing HLA-DP4 (B16cDP4) or expressing DP4 under an IFNγ inducible promoter (B16iDP4). HLA-DP4 transgenic mice were implanted with B16cDP4 or B16iDP4 cells and four days later immunisation commenced with a vaccine containing all seven selected citrullinated peptides. The combination vaccine provided significant tumour therapy in the B16cDP4 model with 40% tumour free survival compared to adjuvant only control (p=0.0149) (**Figure 1A**). Many tumours do not constitutively express MHCII therefore we also examined the tumour therapy in a B16 tumour model where HLA-DP4 is under the control of an IFNγ inducible promoter. The combination vaccine showed reduced efficacy in the B16iDP4 model and did not reach significance with only 20% tumour free survival compared to adjuvant only control (**Figure 1B**). As the 7 peptide combination did not provide strong anti-tumour immunity the contribution of individual peptides was assessed in HLA-DP4 transgenic mice. Immunisation with the combination vaccine demonstrate evidence of IFNγ responses only to citrullinated matrix metalloproteinase-21 (citMMP21) peptide (**Figure 1C**).

To determine if the dominant response to citMMP21 was due to strong HLA-DP4 binding, all seven peptides were screened for binding to HLA-DP4 in a competition assay against a known HLA-DP4 peptide from Hepatitis B. **Figure 1D** shows that the Hepatitis B peptide competes equally with itself showing a result of 50% maximal binding when equal quantities of reference and competitor peptide are used. As a negative control, two peptides from collagen and fibrinogen known not to bind to HLA-DP4 (25) show no competition with the Hepatitis B reference peptide. All seven citrullinated peptides show competition with the Hepatitis B peptide. Competition with citrullinated elongation factor 1 alpha (citELO), trophoblast glycoprotein-like (cit5T4), cytochrome p450 1A2 (citCp450), glutamate receptor ionotrophic (citGRI) and matrix metalloproteinase-21 (citMMP21) peptides all show 40-50% binding of the reference Hepatitis B peptide suggesting that they have similar binding affinity to the Hepatitis B peptide. Competition with the citrullinated serine/threonine protein phosphatase (citSeThrePho) and 26S protease regulatory subunit 10B (cit26S-10B) peptides reveal increased inhibition of the Hepatitis B reference peptide with a reduction to 30-35% binding (p=0.0014 and p=0.0003 respectively), indicating that these two peptides have a stronger binding affinity for HLA-DP4 compared to the Hepatitis B peptide. Thus, we can confirm that the seven selected citrullinated peptides all bind efficiently to HLA-DP4. This suggested that the dominant response to citMMP21 may be due to recognition of this peptide/HLA-DP4 combination by a higher affinity TCR.

### Citrullinated Peptides Eluted From Tumours Elicit CD4 Th1 Immune Responses

**Figure 2:**
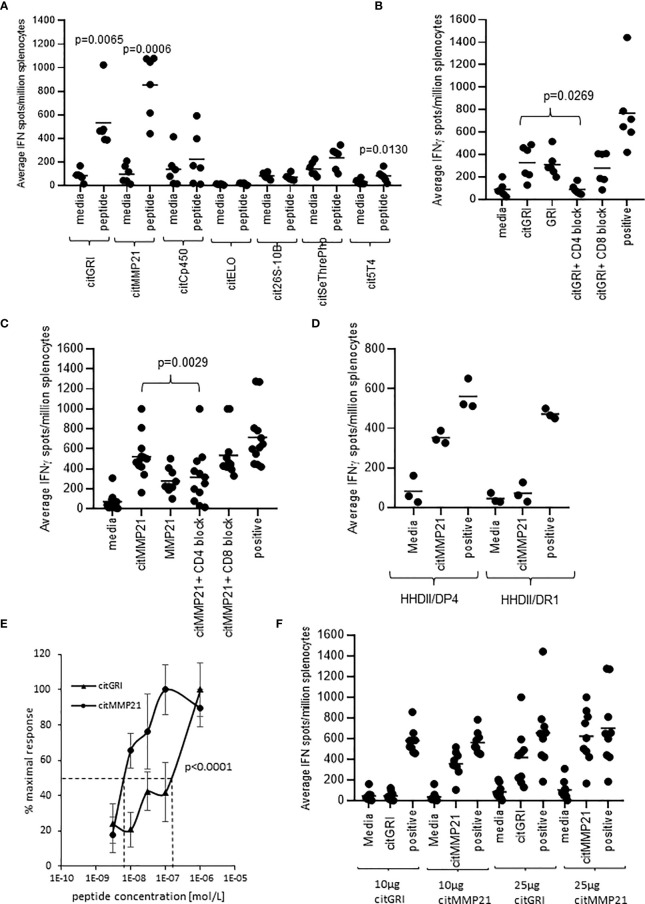
Eluted citrullinated peptides show strong CD4 Th1 immune responses. HLA-DP4 **(A)** transgenic mice were immunised with 25ug of individual citrullinated peptides on days 1, 8 and 15 and immune responses specific to the citrullinated peptides were monitored on day 21 by IFNγ Elispot assay and compared to control. Immune responses in mice immunised with 25ug citGRI **(B, E)** or citMMP21 **(C–E)** were analysed in the presence of CD4 or CD8 blocking antibody **(B, C)** and titrating quantities of peptide **(E)**. Immune responses to citMMP21 were compared in HHDII/DP4 and HHDI/DR1 mice **(D)**. Comparison of 25ug and 10ug immunising peptide dose for citGRI and citMMP21 peptides **(F)**. Results are shown from a representative example **(D, E)** or at least two independent experiments in which n=3.

To assess the immunogenicity of the seven peptides, HLA-DP4 transgenic mice were immunised with 25µg of single peptides in combination with CpG and MPLA adjuvants. Immune responses were analysed in isolated splenocytes by IFNγ ELISpot assay. IFNγ responses were detected against three of the seven peptides compared to media negative control (**Figure 2A**). Mice immunised with the citMMP21 peptide showed strong IFNγ responses (p=0.0006) as had been seen in the combination vaccine. Of interest was that immunisation with citGRI peptide also showed strong IFNγ responses to the immunising peptide (p=0.0065) which was lost in the combined vaccine. This suggests that there are subdominant responses that are perhaps influenced by the other peptides included in the combination vaccine. To determine if responses were mediated by CD4 or CD8 T cells, responses were analysed in the presence of CD4 or CD8 blocking antibodies. The response to citGRI was efficiently blocked in the presence of the CD4 blocking antibody (p=0.0269) but not the CD8 antibody confirming it to be CD4 mediated (**Figure 2B**). However, responses showed cross reactivity to the unmodified GRI peptide. The response to citMMP21 was reduced by the CD4 blocking antibody (p=0.0029) to the level seen to the unmodified MMP21 peptide (**Figure 2C**). No significant blocking was seen with the CD8 blocking antibody suggesting the response to the citMMP21 epitope is CD4 mediated (**Figure 2C**). To further investigate this, citMMP21 was assessed for responses in HHDII/DR1 transgenic mice that have the same MHCI allele but different MHCII. No responses were seen in the HHDII/DR1 mice implying that the response in the HHDII/DP4 mice is likely CD4 mediated (**Figure 2D**). The avidity of the immune responses to citMMP21 (10^-8^M) was significantly higher than the avidity of the response to citGRI (10^-7^M) (p<0.0001) (**Figure 2E**). To identify if the T cell response was dose dependent, mice were immunised with 10µg of peptides as used in the combined vaccine, in these experiments only the response to citMMP21 was observed whereas at the 25µg peptide dose a response to both peptides was observed (**Figure 2F**). These results suggest that in the face of similar MHCII binding affinity, the TCRs recognising citGRI/HLA-DP4 combination are potentially lower affinity requiring high levels of peptide for stimulation and explaining its sub dominance in the combination.

### Citrullinated Peptides Eluted From Tumours Elicit CD4 IL-10 Regulatory Immune Responses

**Figure 3:**
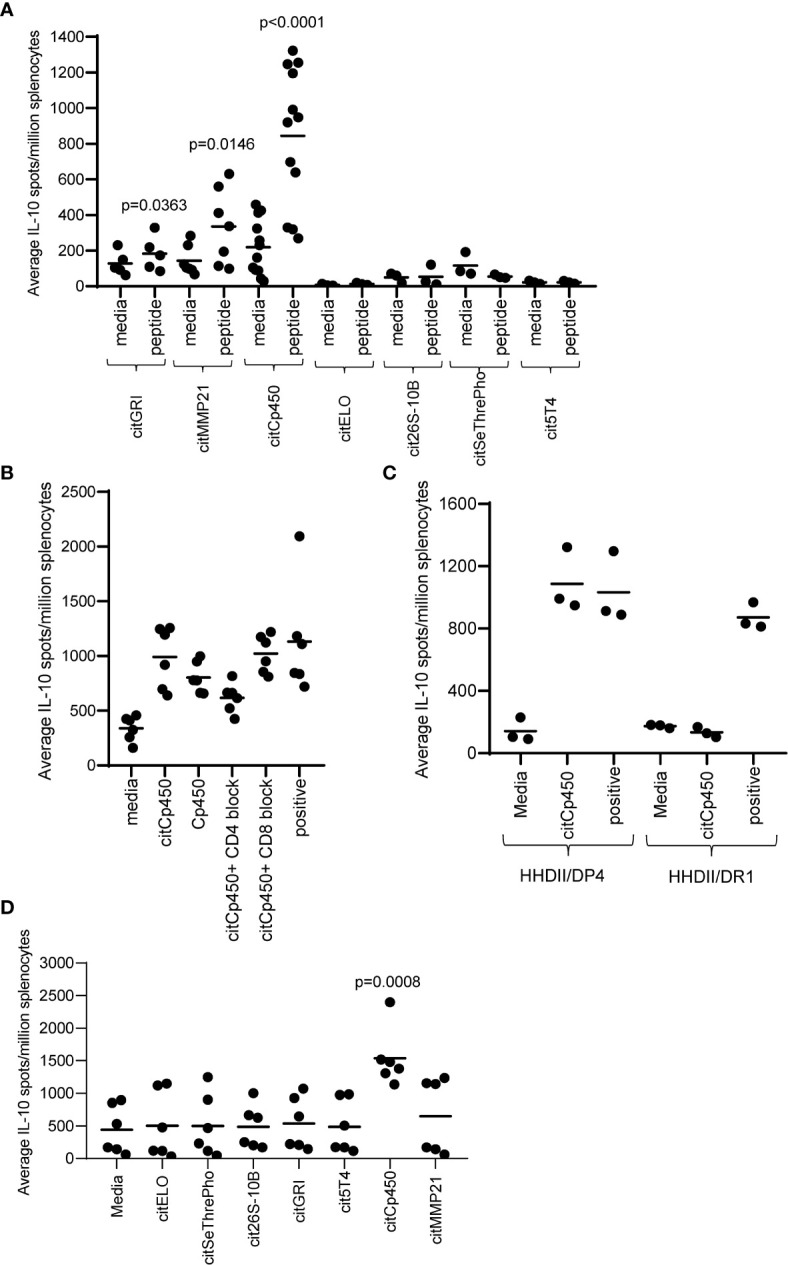
Eluted citrullinated peptides show CD4 IL-10 regulatory immune responses. HLA-DP4 **(A)** transgenic mice were immunised with 25ug of individual citrullinated peptides on days 1, 8 and 15 and immune responses specific to the citrullinated peptides were monitored on day 21 by IL-10 Elispot assay and compared to control. Immune responses in mice immunised with 25ug citCp450 were analysed in the presence of CD4 or CD8 blocking antibody **(B)**. IL-10 responses to citCp450 were compared in HHDII/DP4 and HHDI/DR1 mice **(C)**. Analysis of IL-10 responses from vaccination with the 7 peptide combination vaccine **(D)**. Results are shown from a representative example **(C)** or at least two independent experiments in which n=3.

The absence of IFNγ responses detected to four peptides does not necessarily mean that they do not activate specific T cells. It is possible that the T cells activated do not produce IFNγ therefore we also analysed the mice for the production of IL-10 to these specific peptides. **Figure 3A** shows that citGRI and citMMP21 also stimulate some IL-10-producing cells which is not unusual from a strong Th1 response (p=0.0363 and p=0.0146 respectively). Interestingly, citCp450 stimulated a strong IL-10 response in the absence of any detectable IFNγ response (p<0.0001) and even with the strong Th1 adjuvants CpG/MPLA. Similar to the citGRI and the citMMP21 responses, the IL-10 response induced by citCp450 showed a reduction in response to the citrullinated peptide in the presence of the CD4 blocking antibody suggesting it is CD4 mediated, however this did not reach significance (**Figure 3B**). To confirm if responses were CD4 mediated they were also assessed in the HHDII/DR1 mouse model compared to HHDII/DP4 mice. **Figure 3C** shows that responses fail to be stimulated in HHDII/DR1 mice suggesting that this response is CD4 mediated in the HHDII/DP4 mice.

None of the other peptides showed any evidence of IL-10 responses. In addition, citCp450 stimulated IL-10 responses when delivered as part of the combination vaccine suggesting this response is not affected by the other epitopes in the combination vaccine but may result in an impaired anti-tumour response (**Figure 3D**).

### Individual Citrullinated Peptide Vaccination Mediates Efficient Tumour Therapy

**Figure 4:**
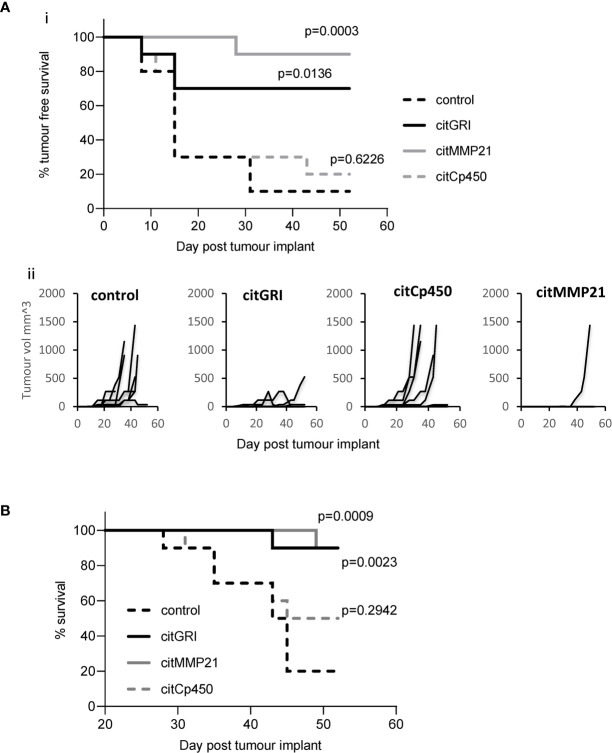
Individual peptide vaccination mediates efficient tumour therapy. HLA-DP4 transgenic mice were challenged with B16 cells constitutively expressing DP4 and four days later mice were immunised with 25ug citGRI, citMMP21 or citCp450 peptides and tumour growth **(Aii)** monitored. Survival presented as tumour free survival over time **(Ai)** and overall survival up to day 50 **(B)** Results shown are representative of at least two independent studies in which n=10/group.

Vaccination with three of the identified citrullinated peptides has been shown to stimulate CD4 T cell responses. As part of a combination vaccine these peptides failed to provide convincing tumour therapy. They were therefore assessed for tumour therapy as individual peptide vaccines. Mice were implanted with B16cDP4 cells followed by immunisation with each of the citrullinated peptides. Mice treated with citGRI demonstrates significantly enhanced tumour therapy with 70% tumour free survival (p=0.0136) as well as significant overall survival at day 50 (p=0.0023) compared to unimmunised control (**Figures 4A, B**). citMMP21 also provided efficient tumour therapy with 90% tumour free survival (p=0.0003) (**Figure 4A**) and in line with its dominant response it also induced strong overall survival at day 50 (p=0.0009) (**Figure 4B**). Immunisation with citCp450 showed tumour growth similar to control with only 20% tumour free survival which was not significant over control (**Figures 4A, B**). This data suggests that the citrullinated peptides identified on the tumour surface that are capable of stimulating a Th1 response can be harnessed for vaccine mediated immune therapy. However, peptides identified that stimulate predominantly a regulatory IL-10 response such as citCp450 fail to mediate tumour therapy.

### CitCp450 Mediated IL-10 Response Is Not Exclusively Responsible for the Loss of Tumour Therapy With the Combination Vaccine

**Figure 5:**
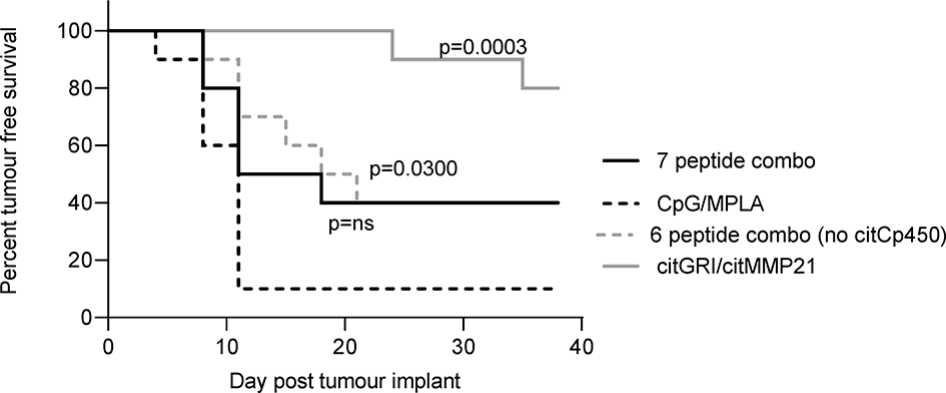
citCp450 is not responsible for lack of tumour therapy of the combination vaccine. HLA-DP4 transgenic mice were challenged with B16 cells constitutively expressing DP4 and four days later mice were immunised with 10ug each of citGRI and citMMP21 duo compared to the seven peptide cocktail or a six peptide cocktail without the citCp450 peptide. n = 10/group.

The anti-tumour response to citMMP21 was significantly stronger when administered on its own than when used in a combination vaccine suggesting that despite its immunodominance it is still affected by the other peptides within the combined vaccine. The IL-10 response to citCp450 was detected within the combination vaccine as well as from the single peptide vaccine. The presence of an IL-10 response in the combination vaccine has the potential to exert an inhibitory effect over the Th1 responses in the same combination. We therefore examined the tumour therapy mediated by the combination vaccine excluding citCp450. The combination vaccine demonstrated 40% tumour free survival but this did not reach significance compared to the adjuvant only control (p=0.0918). Exclusion of citCp450 from the combination shows little difference in tumour therapy compared to the combination of all 7 peptides, both with 40% tumour free survival, but does show a significant effect compared to adjuvant only control (p=0.0300) (**Figure 5**). This data implies that although the IL-10 responses stimulated by this peptide do not mediate tumour therapy, they may play a small role in inhibiting tumour therapy but that other factors such as T cell affinity/avidity are more relevant.

Data included in this study demonstrates the identification of citrullinated peptides presented on tumour MHCII by peptide elution and mass spectrometry and that these can be targeted for tumour immunotherapy. We provide the first evidence in a mouse model that tumours present citrullinated epitopes that can stimulate both Th1 and regulatory CD4 responses and suggest that characterisation of responses elicited by epitopes identified by peptide elution is necessary to optimise candidate selection for tumour therapy.

## Discussion

Citrullinated peptide epitopes are unable to be reliably predicted from genomic analysis or *via* conventional MHC binding algorithms. However, HLA elution and mass spectrometry analysis of the HLA ligandome provides a useful tool for the analysis of post-translationally modified peptides. We demonstrate in this study that citrullinated peptide epitopes can be detected on the surface of *in vivo* grown murine B16 tumour cells expressing HLA-DP4 through conventional HLA ligandome analysis. One of the advantages of the HLA ligandome analysis of tumour samples is the identification of many potential novel presented peptides that could act as targets for tumour therapy (13, 28). Indeed several groups have exploited this and investigated the use of eluted peptide epitopes in immunotherapy in both preclinical and clinical studies (29–31). The majority of these studies focus primarily on the elution of MHCI binding peptides and the stimulation of CD8 T cell responses. It is however encouraging to show that vaccines comprised of epitopes identified by peptide elution can stimulate responses *in vivo* in humans (29, 32). It is likely that each tumour presents a very different repertoire of peptide epitopes and therefore identifying a set of peptides for a universal vaccine is challenging. To facilitate this most studies have examined peptides from a large number of tumour samples to compile a vaccine candidate that is applicable to many individuals. This approach also has the potential to overcome the issue of peptide binding competition if peptides are selected from a range of HLA alleles.

A recent study by Katayama et al. has demonstrated an association of increased PAD2 expression in breast cancer with increased citrullinated peptide presentation on MHCII proposing citrullinated peptides as neoantigen candidates (18). Indeed, elevated PAD expression can lead to an increase in citrullinated peptides, but this increase would also be dependent on the levels of PAD activity which is associated with intracellular calcium levels as well as the level of MHCII expression which may depend upon the presence of an inflammatory environment and also enhancement of alternate routes of MHCII epitope presentation such as autophagy.

In this study, identified peptides were selected from a single HLA allele and showed an equal distribution of lengths between 8 to 20 amino acids rather than the classical gaussian distribution centred around 15 to 16 amino acids thus suggesting perhaps the total quantity of peptides sequenced may be low. With respect to identification of MHCII epitopes, the CD4 T cell repertoire to high affinity self-peptides is often deleted, tolerised or polarised to a regulatory repertoire as a mechanism to prevent autoimmunity (33). In spite of this we saw evidence of strong immune responses to three of the seven high affinity eluted peptides helping to confirm these as valid citrullinated peptides although further analysis should be performed to validate these as citrullinated sequences by mass spectrometry. The levels of these citrullinated peptides and their native counterparts expressed in tumour should also be confirmed by methods such as multiple reaction monitoring mass spectrometry (MRM-MS) with stable isotope dilution (SID) and this may also provide explanation as to why some peptides stimulated pro-inflammatory responses and some anti-inflammatory responses. However, response identification to the three citrullinated peptides suggests they are likely genuine and repertoires were not deleted or tolerised. Recent evidence from Cebula et al. suggests that not all CD4+ T cells to higher affinity self-epitopes are deleted and repertoires do exist in the periphery but these are kept under control by peripheral regulatory T cell populations (34). The lack of responses detected in this study to four of the identified peptides suggests that these may not have been valid epitopes or that repertoires may be deleted. However, we haven’t examined the full range of cytokines that represent all T helper subsets so further studies would be required to conclude that these repertoires were deleted or to validate the citrullinated peptide identification by mass spectrometry. Identification of an IL-10 response in the absence of any IFNγ to citCp450 indicates that in addition to the Th1 responses a potential regulatory repertoire exists in the mice to a citrullinated self-antigen that is presented by B16 tumour. The regulatory phenotype of the citCp450 epitope was also highlighted by the failure to provide any tumour therapy after peptide vaccination although it did not appear to make tumour growth more aggressive. Thus supporting the hypothesis for the existence of peripheral regulatory cells and demonstrating the antigen specificity of this response. To further characterise the responses detected in this study as Th1 and regulatory, additional cytokine analysis would be required and TCR analysis to determine the clonality of the responses as well as recognition of both citrullinated and native alternative core sequence presented by HLA-DP4. Citrullination occurs in thymic dendritic cells and may be partially responsible for T cell repertoire selection. As citrullination is mediated by PAD enzymes which require high levels of calcium, it can only occur in double membrane vesicles such as nuclei or autophagosomes (17, 35). The latter can be sampled by the MHCII processing pathway for presentation of epitopes on the cell surface (17, 36). Most cells do not constitutively express MHCII but it can be induced by IFNγ (37). Thus only cells in which autophagy is induced by stress and which are in an inflammatory environment will express citrullinated epitopes and be targets for these T cells. To restrict autoimmunity perhaps polarised Tr1 responses secreting IL-10 are induced. Rheumatoid arthritis (RA) is characterised by anti-citrullinated antibody responses of the IgG subclass which requires T cell help (38). RA is mainly restricted to individuals expressing shared HLA-DR alleles (39). Perhaps enhanced binding of citrullinated epitopes to these alleles allows stimulation of subdominant Th1 responses. It is worth noting that the Th1 responses in this study also showed cross reactivity to the native sequences which could be of concern should the native sequences be presented by MHCII. Although we saw no evidence of toxicity, possibly due to the restricted expression of MHCII under normal conditions, further work should determine presentation of the native sequence by tumours and normal tissues.

Despite identification of citrullinated peptides presented by the B16 tumour that can stimulate Th1 responses, tumours grew and progressed suggesting the regulatory tumour microenvironment efficiently subdues these responses. However, we demonstrate that vaccination with either citGRI or citMMP21 stimulated strong Th1 IFNγ responses in mice that overcame any peripheral or tumour mediated regulation to elicit efficient tumour therapy. The significant role of CD4 responses in tumour therapy is becoming clearer with several groups highlighting the importance of CD4 T cells in immunotherapy regimes (8–10). We have demonstrated in this study and in previous work the potency of Th1 responses and provide further evidence for their role in tumour therapy (11, 19, 20). This provides further evidence to support an important role for strong tumour specific Th1 responses in tumour therapy. The variety of immune phenotypes shown among the selected eluted peptides in this study supports the hypothesis that tumour presented MHCII epitopes can be specific for both regulatory and proinflammatory responses, a phenomenon also seen by others (8, 10, 40). The combination of all seven selected eluted peptides into a single vaccine provided little beneficial tumour therapy whereas immunisation with only the peptides capable of eliciting Th1 responses generated efficient tumour therapy of the aggressive B16 model. Initially we hypothesised that the presence of the regulatory cytochrome p450 response was responsible for the lack of tumour therapy. Studies with a combination lacking this peptide however demonstrated that this response had a small effect but was not responsible for the total lack of therapy seen with the combination. As all the peptides in the combined vaccine have similar binding affinities to MHCII they may compete for binding and present a lower dose of each individual peptide on antigen presenting cells. A phenomenon similar to this is seen with other multiepitope vaccines restricted through a single HLA allele where often response to a particular epitope dominates (41, 42).

In addition to peptide/MHC binding playing a role in epitope presentation, the avidity of the TCR interaction with peptide/MHC complex also plays an important part in T cell recognition. The assignment of avidity is determined functionally by the amount of peptide required for activation of effector function. It is a measure of the overall strength of the interaction between a T cell and a target cell. T cells recognising high affinity self epitopes with highest avidity are often deleted or anergised leaving a lower avidity repertoire available that relies on higher levels of peptide presentation for activation. Indeed, the avidity of the T cell responses to citGRI was significantly lower than the avidity of the citMMP21 response and lower doses of citGRI peptide failed to stimulate an immune response. It is widely accepted that the generation of high frequency T cell responses is not necessarily an indication of the induction of an effective immune response. It is apparent from previous published work that T cell functional avidity is a better indicator of clinical response (43–47). The term functional avidity is often confused with affinity. Affinity is most often classified as a measure of the strength of binding of the peptide MHC molecule to the T cell receptor (TCR) whereas functional avidity is a measure of the combination of stimulation *via* TCR, co-stimulatory molecules, adhesion molecules and cytokines and is indicative of the overall strength of interaction between T cell and target and its functional outcome (48). In both viral infection and tumour models, only high avidity T cells mediate viral clearance and tumour eradication (49–51). During the generation of an immune response *in vivo* T cells can show a range of functional avidities both at the clonal and polyclonal level. Although avidity has been shown to be important in both viral and tumour settings, the mechanisms by which high and low avidity T cells are generated *in vivo* remains unclear as the TCR cannot undergo somatic hypermutation. It has been demonstrated *in vitro* that culturing of T cells in the presence of high or low dose of antigen leads to polarisation of low and high avidity responses respectively. Presentation of varying amounts of peptide within the thymus may also select for T cells with differing affinities to self-antigens.

From this study we conclude that HLA ligandome analysis provides a powerful tool for the identification of post-translationally modified epitopes presented by tumours. We provide evidence in our mouse model that tumours present epitopes capable of stimulating diverse immune responses consisting of both Th1 and regulatory responses and propose this as another way by which tumours modulate the immune environment. We suggest that citrullinated peptide identification *via* elution from MHCII can yield strong targets for tumour immunotherapy but careful consideration and characterisation of the choice of MHCII epitopes to include in any immune therapies should be employed to avoid effects of immune competition and regulation.

## Data Availability Statement 

Materials are available upon reasonable request from the corresponding author. The MS dataset presented in this study is available via ProteomeXchange with identifier PXD029029.

## Ethics Statement 

All animal work was carried out under a UK Home Office approved project license and in accordance with EU Directive 2010/63/EU and with approval of Nottingham Trent University ethics committee.

## Author Contributions

LD and VB directed the study. PS, AM, and KC performed experiments and analysed data. RM provided reagents. VB and LD designed experiments, analysed the data and wrote the paper. All authors contributed to the article and approved the submitted version.

## Funding

This work was funded by Scancell Ltd. AM was financed through a stipend from the Bosch Research Foundation.

## Conflict of Interest

LD is a director and shareholder in Scancell Ltd. PS, KC, RM and VB are employees of Scancell Ltd.

The authors declare that this study received funding from Scancell Ltd. The funder had the following involvement with the study: study design, collection, analysis, interpretation of data, the writing of this article.

## Publisher’s Note

All claims expressed in this article are solely those of the authors and do not necessarily represent those of their affiliated organizations, or those of the publisher, the editors and the reviewers. Any product that may be evaluated in this article, or claim that may be made by its manufacturer, is not guaranteed or endorsed by the publisher.

## Abbreviations

IFNγ, Interferon γ; MHC-II, Major histocompatibility complex class II; MHC-I, Major histocompatibility complex class I; HLA, Human leukocyte antigen; TCR, T cell receptor; TME, tumour microenvironment; PAD, peptidyl arginine deaminase; PTM, post translational modification; FCS, Fetal calf serum; LPS, Lipopolysaccharide; Th1, T helper 1; WT, wildtype; ELISA, Enzyme linked immunosorbent assay; ELISpot, Enzyme linked immunosorbent spot; PBMC, Peripheral blood mononuclear cell; TIL, tumour infiltrating lymphocyte; IL-10, Interleukin 10; MPLA, monophosphoryl lipid A; HRP, horseradish peroxidase; ANOVA, analysis of variance; RA, rheumatoid arthritis; IEDB, Immune Epitope Database.
