# Supplementary material for: Citrullinated Epitopes Identified on Tumour MHC Class II by Peptide Elution Stimulate Both Regulatory and Th1 Responses and Require Careful Selection for Optimal Anti-Tumour Responses
Source: Front Immunol. 2021 Nov 9;12:764462. doi: 10.3389/fimmu.2021.764462 (PMC8630742; doi:10.3389/fimmu.2021.764462)
Supplement: Supplementary file 4 [file Table_4.docx]

**Supplementary Table 4.** Sequences identified by mass spectrometry and predicted native sequence binding scores to HLA-DP4.

| **Peptide sequence** | **Length (aa)** | **Protein** | **Protein accession number** | **Gene name** | **Predicted HLA-DP4 binding score** | **Predicted core binding region** |
| --- | --- | --- | --- | --- | --- | --- |
| ALLLRVYAxGxKQ | 13 | Adenosine 3'-phospho 5'-phosphosulfate transporter 1 | Q91ZN5 | Slc35b2 | 41.5 | LLRVYA**R**G**R** |
| LPPGIRWPxRNRSSLRRRWLHH | 22 | WSC domain-containing protein 1 | Q80XH4 | Wscd1 | 48.5 | RSSLRRRWL  IRWP**R**RNRS |
| KVKLVVxYTPKVLEEMESR | 19 | Protein lin-7 homolog C | O88952 | Lin7c | 26.45 | VV**R**YTPKVL  YTPKVLEEM |
| AQREEExSQADSALYQMQLETEKER | 25 | Centrosome-associated protein CEP250 | Q60952 | Cep250 | 48.5 | ALYQMQLET  LYQMQLETE |
| TVEDxFDQQKNDYDQLQKA | 19 | Desmoplakin | E9Q557 | Dsp | 72.0 | TVED**R**FDQQ |
| LEIYxYTSFVPFTIPHSTTR | 20 | Cytochrome P450 1A2 | P00186 | Cyp1a2 | 0.41 | IY**R**YTSFVP  Y**R**YTSFVPF |
| VAIAxAIL | 8 | ATP-binding cassette sub-family B member 7, mitochondrial | Q61102 | Abcb7 | ND | ND |
| NGQTxEHALLAYTLGVKQLIVGVNK | 25 | Elongation factor 1-alpha 1 | P10126 | Eef1a1 | 26.0 | T**R**EHALLAY |
| GMExVWCVASLxGSNNVALGYDE | 23 | Coatomer subunit beta' | O55029 | Copb2 | 59.0 | **R**VWCVASL**R**  L**R**GSNNVAL |
| VPxHPAATSWYEEFxRLYDMVPCV | 24 | F-box/WD repeat-containing protein 5 | Q9QXW2 | Fbxw5 | 26.5 | F**R**RLYDMVP  SWYEEF**R**RL |
| MLxCASGAELxQPPRDVPPDAR | 22 | Trophoblast glycoprotein-like | Q8C013 | Tpbgl | 61.50 | L**R**CASGAEL |
| IKxCSEFESxLEGYSKELEMFRKRE | 25 | Dynein heavy chain 3, axonemal | Q8BW94 | Dnah3 | 40.5 | **R**LEGYSKEL  K**R**CSEFES**R** |
| YLLPILVLFLAYYYYSTNEEFx | 22 | Corticosteroid 11-beta-dehydrogenase isozyme 1 | P50172 | Hsd11b1 | 6.4 | LVLFLAYYY  FLAYYYYST |
| FxTIHQACKLICxK | 14 | Zinc finger and BTB domain-containing protein 8A | Q9CWH1 | Zbtb8a | 44.5 | **R**TIHQACKL  HQACKLIC**R** |
| GxHNGIDGLIPHQYIVVQDTEDG | 23 | SLIT-ROBO Rho GTPase-activating protein 2 | Q91Z67 | Srgap2 | 43.0 | IPHQYIVVQ  LIPHQYIVV |
| ExKRARGISPIVF | 13 | YTH domain-containing protein 1 | E9Q5K9 | Ythdc1 | 55.0 | RARGISPIV  KRARGISPI |
| VFDWIRKERNQYGEVRVxFNTYFFR | 25 | Matrix metalloproteinase-21 | Q8K3F2 | Mmp21 | 17.0 | RV**R**FNTYFF  EVRV**R**FNTY |
| LPECIDALTVSLESVQSxAAWR | 22 | Nesprin-2 | Q6ZWQ0 | Syne2 | 50.5 | DALTVSLES  ALTVSLESV |
| MQRMHNPEREAKKADxISRSKTF | 23 | PR domain zinc finger protein 10 | Q3UTQ7 | Prdm10 | 91.5 | RMHNPEREA  KKAD**R**ISRS |
| QKTGTAEMSSILEExILGADTSVD | 24 | ATP synthase subunit alpha, mitochondrial | Q03265 | Atp5f1a | 60.5 | SSILEE**R**IL  ILEE**R**ILGA |
| LGWSAPVAISRPLxACQM | 18 | Ubiquitin carboxyl-terminal hydrolase 37 | Q8C0R0 | Usp37 | 28.5 | PVAISRPL**R**  VAISRPL**R**A |
| EPKSSCYNTHEKxIYQSNMLNxYLI | 25 | Glutamate receptor ionotropic, NMDA 2B | Q01097 | Grin2b | 31.0 | K**R**IYQSNML  YQSNMLN**R**Y |
| FLSCFSPEYRxITL | 14 | Synaptic vesicle glycoprotein 2A | Q9JIS5 | Sv2a | 20.5 | FLSCFSPEY |
| KRDVTLEASxESSKPxAVLKPx | 22 | Serine/threonine-protein phosphatase 2A 55 kDa regulatory subunit B gamma isoform | Q8BG02 | Ppp2r2c | 65.50 | VTLEAS**R**ES  LEAS**R**ESSK |
| MDEIDAIGGxRFSEGTSADREIQ | 23 | 26S protease regulatory subunit 10B | P62334 | Psmc6 | 56.00 | IGG**R**RFSEG |

x = citrulline, bold = arginine that is citrullinated

The MHCII binding predictions were made on 5/20/2020 using the IEDB analysis resource Consensus tool [^24^](https://scancell-my.sharepoint.com/personal/victoriabrentville_scancell_co_uk/Documents/Work/Reports%20and%20stuff/Scancell%20papers/Eluted%20peptides/JITC%20submission/Eluted%20peptide%20paper%20Mar%202021(ld)%20(KC)vb%20310321.docx#_ENREF_24) [^25^](https://scancell-my.sharepoint.com/personal/victoriabrentville_scancell_co_uk/Documents/Work/Reports%20and%20stuff/Scancell%20papers/Eluted%20peptides/JITC%20submission/Eluted%20peptide%20paper%20Mar%202021(ld)%20(KC)vb%20310321.docx#_ENREF_25).
